# Mapping risk of plague in Qinghai-Tibetan Plateau, China

**DOI:** 10.1186/1471-2334-14-382

**Published:** 2014-07-10

**Authors:** Quan Qian, Jian Zhao, Liqun Fang, Hang Zhou, Wenyi Zhang, Lan Wei, Hong Yang, Wenwu Yin, Wuchun Cao, Qun Li

**Affiliations:** 1 State Key Laboratory of Pathogen and Biosecurity, Beijing, China; 2 Institute of Health Service and Medical Information, Beijing, China; 3 Chinese Center for Disease Control and Prevention, Beijing, China

## Abstract

**Background:**

Qinghai-Tibetan Plateau of China is known to be the plague endemic region where marmot (Marmota himalayana) is the primary host. Human plague cases are relatively low incidence but high mortality, which presents unique surveillance and public health challenges, because early detection through surveillance may not always be feasible and infrequent clinical cases may be misdiagnosed.

**Methods:**

Based on plague surveillance data and environmental variables, Maxent was applied to model the presence probability of plague host. 75% occurrence points were randomly selected for training model, and the rest 25% points were used for model test and validation. Maxent model performance was measured as test gain and test AUC. The optimal probability cut-off value was chosen by maximizing training sensitivity and specificity simultaneously.

**Results:**

We used field surveillance data in an ecological niche modeling (ENM) framework to depict spatial distribution of natural foci of plague in Qinghai-Tibetan Plateau. Most human-inhabited areas at risk of exposure to enzootic plague are distributed in the east and south of the Plateau. Elevation, temperature of land surface and normalized difference vegetation index play a large part in determining the distribution of the enzootic plague.

**Conclusions:**

This study provided a more detailed view of spatial pattern of enzootic plague and human-inhabited areas at risk of plague. The maps could help public health authorities decide where to perform plague surveillance and take preventive measures in Qinghai-Tibetan Plateau.

## Background

Plague, caused by *Yersinia pestis,* is a rapidly progressing, highly infectious, and highly feared disease that is likely to be fatal without prompt antibiotic treatment [1]. Plague is maintained among wild rodents in distinct geographic foci showing a serious threat to humans in Asia, America, and Africa [2]. Although the mechanisms by which plague is maintained between epizootic cycles are not well understood, it is generally accepted that the disease cycles between enzootic infections and occasional epizootic outbreaks among susceptible hosts [3]. Human plague cases are relatively low incidence but high mortality, which presents unique surveillance and public health challenges, because early detection through surveillance may not always be feasible and infrequent clinical cases may be misdiagnosed.

In mainland China, natural plague foci of plague were divided into 12 types according to their primary reservoirs, principal vectors, landscapes and genotypes of *Y. pestis*[4], which were distributed in 19 of total 31 provinces and autonomous regions., The marmots (*Marmota himalayana*) foci in Qinghai-Tibetan Plateau was most active and widespread. The primary host, *M. himalayana* usually hibernates from October and comes out of hibernation from April the next year [5]. *Callopsylla dolabris* and *Oropsylla silantiewi* are known as the principal vectors [4]. The epizootics of plague occurs during the whole period when the rodent hosts are active on the ground, and usually reaches the peak in June and July [5]. Human cases occurred almost every year in the enzootic region [6]. In 2009, an outbreak involving 12 pneumonic plague cases occurred in a remote village of Qinghai Province leading to three deaths [7]. In 2010, cases with bubonic plague and pneumonic plague were reported in Gansu Province and Tibet Autonomous Region.

In mainland China, the natural foci of plague had been discovered and described since 1950, which were plotted out based on administrative boundary. This approach is rather rough and enlarged the potential risk area. Natural foci of plague are related to the particular landscapes which are favorable for a high and stable number of rodent reservoirs and flea vectors of *Y. pestis*[8-10]. Thus, understanding how these host species are geographically distributed in China and relating that information to how effciently each can transmit plague is critical to under-standing the ecology of plague and to recognizing where the greatest threats for people exist in the country.

The “Host Niche Hypothesis” (HNH) postulates that plague distributions are mediated by host distributions, such that the distribution of plague depends on an amalgam of host ranges, and the presence of a particular host species could extend the distributional potential of the pathogen [11]. Thus, by predicting the potential distribution of host animal, the plague risk could be mapped. Topography, vegetation, climate and other environmental factors are thought to influence spatial distibution and temporal dynamics of the host animal [12-18]. Novel spatial modelling methods such as maximum entropy (MAXENT) and the genetic algorithm for rule set production (GARP) require only disease presence data and have been used extensively in the fields of ecology and conservation, to model species distribution and habitat suitability [19]. In this paper, we aimed to predict the potential natural foci of plague in Qinghai-Tibetan Plateau by ecological niche modeling based on environmental parameters derived from remote sensing data and the transmission risk to support our control and prevention.

## Methods

### Study area

**Figure 1:**
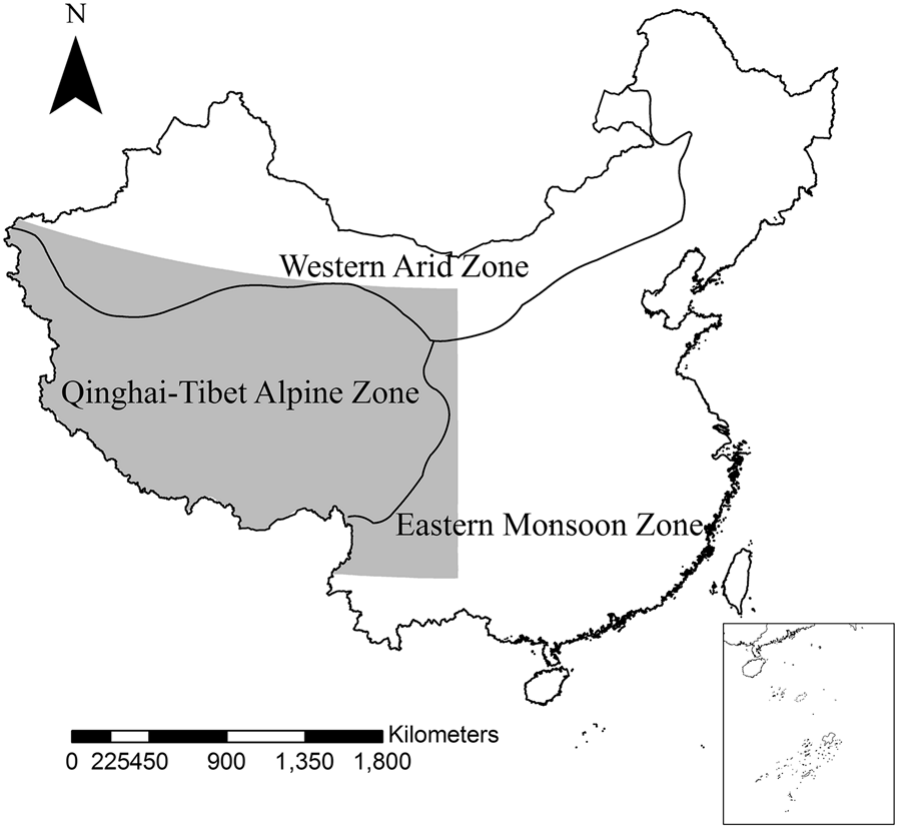
The study area and counties with known natural foci of plague.

The study area stretches from 26°00′12″N to 39°46′50″N and from 73°18′52″E to 104°46′59″E, which covers approximately 3,487,000 km^2^ in Qinghai-Tibetan Plateau (Figure 1), most active and widespread marmots (*Marmota himalayana*) foci area. The altitude is over 3000 m almost everywhere. The main vegetation types are alpine meadow, alpine grassland, alpine desert and Ravine forest [20]. Elevation, temperature, moisture, vegetation and other environmental conditions are various [21].

### Data collection and pre-processing

All plague surveillance data used in this study were derived from the plague surveillance database of ‘Chinese Plague Prevention and Control Management Information System’, a national surveillance network for plague maintained by Chinese Center for Disease Control and Prevention. According to the surveillance from 2007 to 2009, 233 infected himalayan marmots were confirmed by bacteriological tests including microscopy, culture, phagelysis, and mouse inoculation, geocoded by globe positioning system (GPS) or detail address. In addition, 51 human plague cases in the study area from 2004-2010 were collected from Chinese Center for Disease Control and Prevention. As the spatial resolution of enrironmental data was 1 km, we removed duplicated records of the same pixel and got 218 spatially unique points with infected marmots with a spatial precision of 5–10 km (~0.05–0.1°).

Environmental variables supposed to be associated with the existence of natural foci of plague were collected. The distribution of marmot was mainly driven by geomorphology, climate and vegetation [22]. Geomorphology variables, as Elevation, slope, aspect, were derived from SRTM30 (Available from: http://www2.jpl.nasa.gov/srtm/). Climate factors were characterized by Annual average daytime land surface temperature (LSTd) and annual average nighttime land surface temperature (LSTn), composited from eight days MODIS LST products (MOD11A2) from 2004-2009 (Available from: https://lpdaac.usgs.gov/lpdaac/products/modis_products_table). The amount and productivity of vegetation was featured by annual maximum normalized difference of vegetation index (NDVI-) from ten days SPOT-VGT S10 products from 2004-2009 (Available from: http://free.vgt.vito.be/). In addition, land use and cover change (LUCC) revealed the proper grassland and effect of human activity, which was derived from GlobCover Land Cover version V2.2 released by European Space Agency (Available from: http://www.esa.int/esaEO/SEMXB7TTGOF_index_0.html). In addition, Chinese population distribution raster map with 1 km^2^ resolution was used to identify human-inhabited areas with high plague risk by overlayanalysis. This map was obtained from Data Center for Resources and Environmental Sciences Chinese Academy of Sciences.

All data layers were projected in Albers coordinates and generalized to a pixel resolution of ~1 × 1 km for analysis in ENVI 4.5 (ITT Visual Information Solutions, Boulder, CO, USA) and ArcGIS Desktop 9.2 (Environmental Systems Research Institute, Redlands, CA, USA) environment. We also considered the spatial heterogeneity in the whole china using the GeoDetector software [23,24]. The power of determinant (PD) reflects the degree to which a determinant explains the prevalence of the disease.

### Ecological niche modeling

Recently ecological niche modeling (ENM) methods, such as GARP [25] and Maxent [26], have been widely applied in species distribution modeling when only presence data is available for prediction [27,28]. ENM also has been proved to be effective of in applications to questions regarding the geography and ecology of disease transmission [29-31]. In this study, presence-only method Maxent was applied [32,33]. Maxent is a general-purpose method for characterizing probability distributions from incomplete information based on the principle of maximum entropy [26].

Maxent outputs the maximum entropy distribution that satisfies a set of environmental constraints. In place of true absences, Maxent uses background points (pseudo-absences) to evaluate commission [13]. We run the model in the support of the “Maxent” Version 3.3.1 k software [32]. Within Maxent processing, 75% occurrence points (164) were randomly selected for training model, and the rest 25% points (54) were used for model test and validation. 11000 ‘pseudo-absence’ points created by random sampling from areas lacking known presences. To measure the relative contribution of each environmental variable to the predictive model, a jackknife manipulation was performed. Receiver operating characteristic (ROC) analysis was used to evaluate the discrimination ability of models, and to determine the optimal probability cut-off value for classifying the risk areas of plague. We measured Maxent model performance as test gain and test AUC (random 25% testing) for all analyses omitting individual variable, and for each individual variable alone. The AUC is considered as an effective indicator of model performance. The larger the AUC, the highest is the sensitivity rate and the lower is the 1-specificity rate. Usually AUC values of 0 · 5–0 · 7 are taken to indicate low accuracy, values of 0 · 7–0 · 9 indicate useful applications and values of > 0 · 9 indicate high accuracy [34]. The optimal probability cut-off value was chosen by maximizing training sensitivity and specificity simultaneously [35]. All pixels with a probability value at least equal to the optimal value were classified as plague risk area, then we used binomial test based on omission of independent test points and predicted area to determine whether a model predicts the test localities significantly better than random [36].

## Results

**Figure 2:**
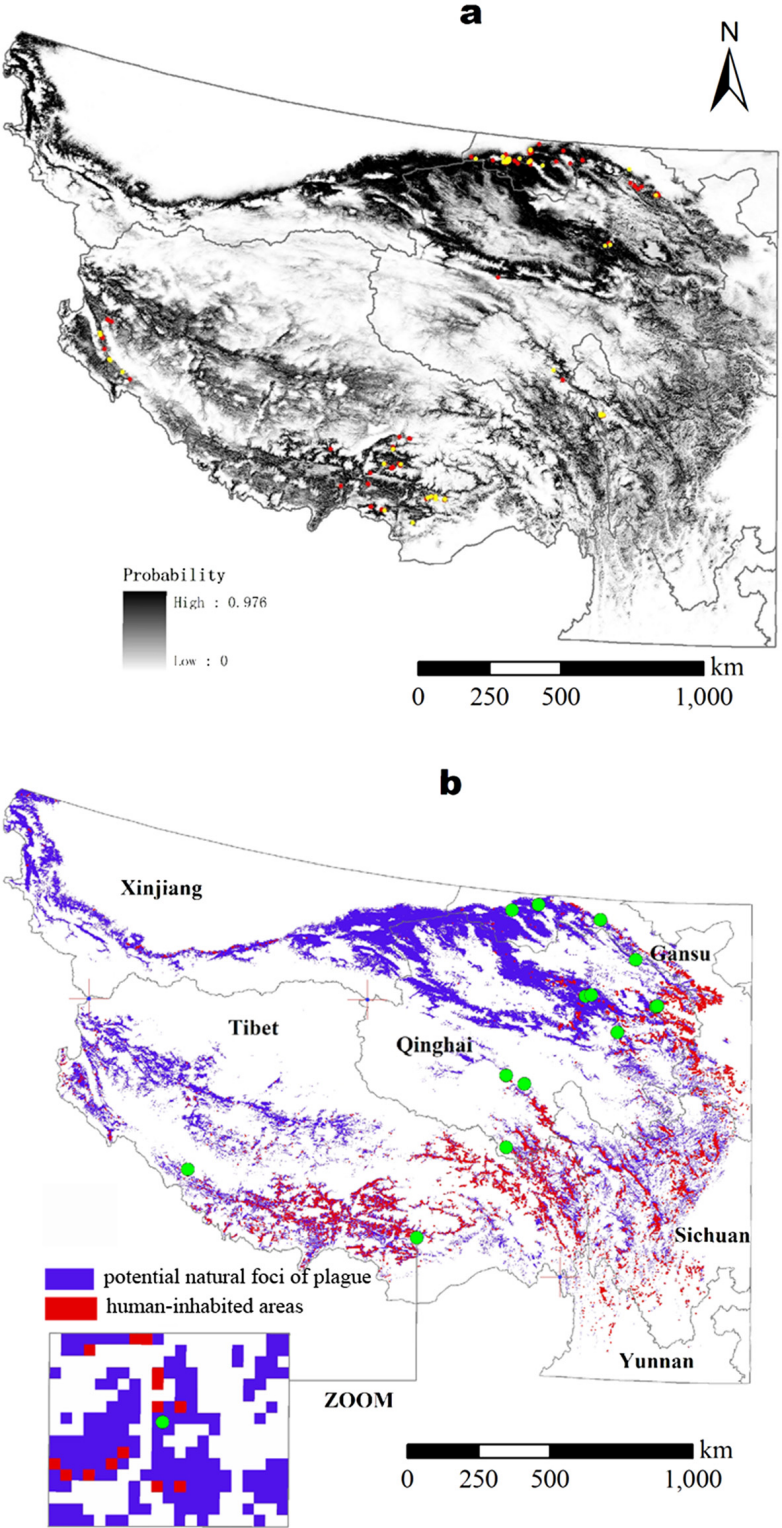
**Risk map of natural foci of plague in Qinghai-Tibetan Plateau. Panel a**: Probability of natural foci of plague. 164 presence points (red circles) for training model and 54 presence points (yellow circles) for testing model are overlaid on the map. **Panel b**: Potential natural foci of plague (blue pixels) and human-inhabited areas (red pixels) in them. 16 sites of human plague cases from 2004 to 2010 (green circles) are overlaid on the map.

The model including all seven environmental parameters was proved to be the most discriminative model attaining the maximum test AUC (test AUC = 0.917) and to be the best fitted model (test gain = 1.5222). All environmental variables appeared to contribute to the model, elevation and land surface temperature had the best explanatory power, with permutation importance 27.5, 15.2 and 32.2, respectively. Meanwhile, they produced the best predictions when used alone and had the most negative effects when omitted from analysis, see Table 1. Thus we selected the all-7-variables model as the final model to construct risk map of marmot distribution. On the basis of ROC analysis of the final model prediction, the optimal risk cut-off value 0.331 was chosen by maximizing training sensitivity and specificity simultaneously. All pixels with a risk value at least equal to this threshold were classified as natural foci where environmental conditions are suitable for plague exist in marmots. 16.5% of the background points were classified in the risk areas. 49 of 54 independent test points were correctly classified (Test omission rate: 9.26%, p <0.0001).The final model was applied to the environmental layers involving NDVI, LSTd, LSTn of 2009, land cover, elevation, slope and aspect. The risk value was predicted for each grid cell in the study area (Figure 2a). The classified natural foci are mostly distributed in Tibet Autonomous Region, Qinghai Province, Gansu Province, the northwest of Sichuan Province, the northwest of Yunnan province and the south of Xinjiang Uygur Autonomous Region (Figure 2b). Large areas without plague presence points used for training and testing models were also predicted as natural foci, such as the northwest corner of Yunnan and the south of Xinjiang.

**Table 1:** Summary of ‘Jackknife analysis ’ used to determine importance of each environmental variable

**Predictor**	**Data type**	**Unit**	**Test gain**	**Test gain**	**Test AUC**	**Test AUC**	**Percent contribution**	**Permutation importance**
			**(variable alone)**	**(variable excluded)**	**(variable alone)**	**(variable excluded)**		
Elevation	Continuous	Metres	0.5668	1.164	0.7848	0.8757	13.3	27.5
LSTd	Continuous	Centigrade	0.3908	1.1436	0.7435	0.876	24.6	15.2
LSTn	Continuous	Centigrade	0.5623	1.2989	0.7405	0.9034	30.2	32.2
NDVI	Continuous	-	0.0159	1.1834	0.5651	0.8829	7.9	14
Slope	Continuous	Degrees	0.0072	1.4199	0.552	0.9025	6.6	5.2
Aspect	Categorical	9 Categories	0.1141	1.441	0.6429	0.9121	11.6	3.5
Land Cover	Categorical	22 Categories	0.1086	1.4787	0.6099	0.9118	5.9	2.5

**Figure 3:**
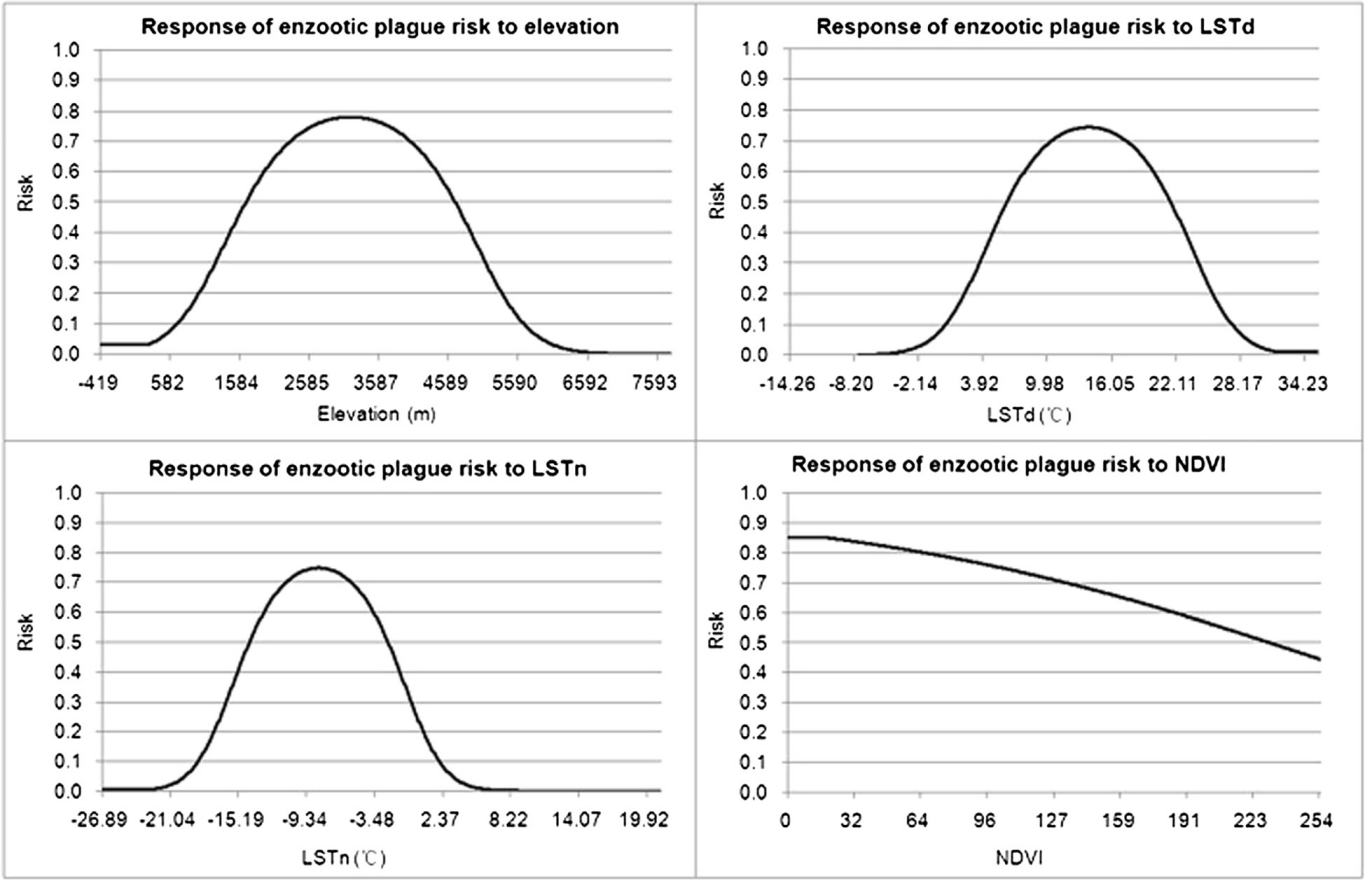
Response of enzootic plague risk to elevation, LSTd, LSTn and NDVI.

**Figure 4:**
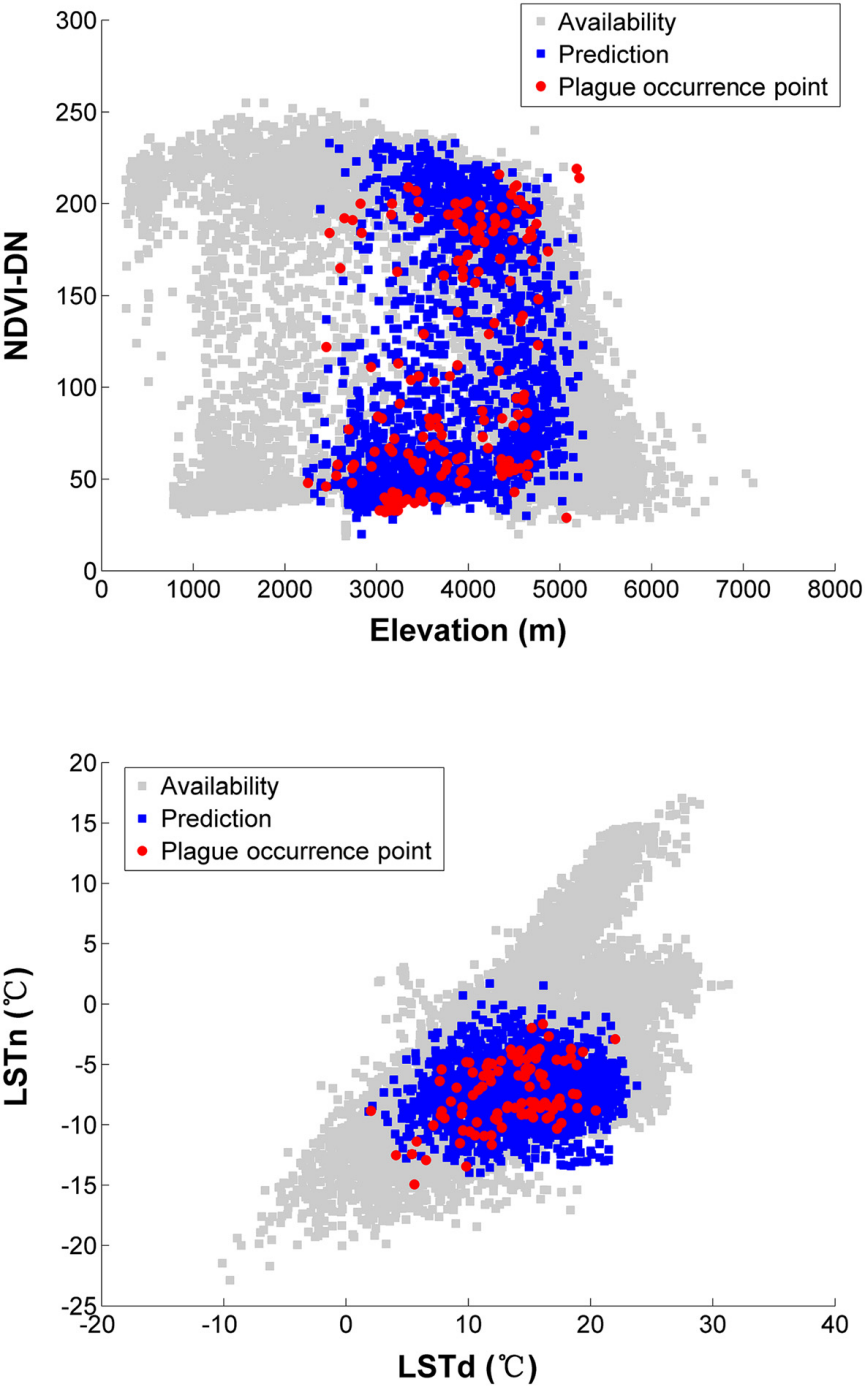
**Visualizations of environmental conditions in the plague-enzootic areas are shown in two-dimensional spaces.** All available environmental conditions in the study area (grey squares), environmental conditions in predicted natural foci of plague (blue squares) and all 218 plague presence points (red circles) are plotted.

By overlying Chinese population distribution map to the risk map created above, we were able to further identify the 16,195 km^2^ human-inhabited areas and about 2,347,000 people at the risk of exposure to enzootic plague in the study area. Most risk population are distributed in the east and south of Qinghai-Tibetan Plateau shown as red pixels in Figure 2b.Several response curves were created to show how the risk of enzootic plague changes as each environmental variable is varied (Figure 3). The risk increases up to an elevation of 3177 m, LSTd of 13.85°C and LSTn of -8.38°C, and then declines as elevation, LSTd and LSTn increases. The risk monotonously decreases as NDVI increases. To visualize and explore the environmental conditions of the plague enzootic areas, two-dimensional scatter plots were developed by using the four key environmental variables described above (Figure 4), The range and the median of elevation, NDVI, LSTd and LSTn of predicted plague enzootic areas are (2240 ~ 5254 m, 3829 m), (0.078 ~ 0.914,0.357), (1.85 ~ 23.80°C,14.30°C) and (-13.97 ~ 1.71°C, -7.25°C), respectively. Environmental conditions which are out of those ranges appear to be important limitations for the distribution of host animal in Qinghai-Tibetan Plateau.

## Discussion

National surveillance network for plague recorded the information of infected marmots as the primary data source of our study. We hypothesis that these geocoded points represented the suitable habitat for plague host animal and fleas that could spread *Yersinia pestis*. In the niche modeling, the relevant environmental variables reflects the distribution of suitable areas of host animals, rather than plague foci or *Yersinia pestis*. This plague-positive host animal extent would be smaller than the whole species on the landscape, but we could not tell the exact proportion or overlap between them because of presence data limitation.

In this study, we applied field surveillance data with GIS/RS-based ecological niche modeling approach to increase our understanding of the suitable habitat of enzootic plague and anticipate the distribution of the natural foci in Qinghai-Tibetan Plateau. The reliable absence data are hardly available in enzootic plague surveillance as the plague-enzootic foci are easily missed during field surveys, thus the “presence-only” ecological niche modeling Maxent was preferred. Independent tests indicated ENM can anticipate the distribution of host animal robustly.

Environmental variables, such as topography, climate and vegetation, can play important roles in natural focal diseases by affecting pathogens directly, or by influencing the distribution and abundance of disease hosts and vectors [37,38]. In this study, all remotely sensed environmental variables appear to contribute positively to the final predictive model. Elevation, LSTd, LSTn and NDVI are likely to be most important factors which influence the distribution of host marmots. The environmental ranges of host marmots were clear revealed in the two-dimensional scatter plots. The environmental conditions of host marmots are proved to be highly diverse. The elevation range of host marmots are various, consistent with Qinghai-Tibetan Plateau [39]. The risk increases up to an elevation of 3177 m and then declines as elevation increase, because in the lower height, forest and thickly grass would be harmful for marmot making doggishness, while in the higher height, there would be lack of food. The marmot preferred temperature between 5°C and 20°C in the daylight. The extremely low temperature below -8.38°C would limit their activities. Extremely low and high temperature of land surface seems negatively affect the distribution of host animals. The temperature of land surface may influence the plague persistence in ‘marmot-flea’ communities in complex ways. We suspected extreme temperature may negatively affect marmot and flea ecology, and blockage of vector fleas [40]. Host marmots were observed and predicted in the sparely vegetated areas, but in our modeling, NDVI variation had no significant limitation in host animal distribution (Figure 3). This result suggested that plague prefer alpine desert/semi-desert grasslands confirmed previously, such as alpine meadow, alpine grassland and alpine shrub [5,39], while habitats with extravagant vegetation seemed to be unsuitable for enzootic plague. This could be due to that luxuriant vegetation obstructs them to protect from predators, and the high subterranean biomass make them hard to burrow underground [41].

Maxent outputs the distribution probability of the host marmots, which was segmented by maximizing training sensitivity and specificity simultaneously. The binarization map reveal massive extent of natural foci of plague in Qinghai-Tibetan Plateau, 16.5% (575,355 km^2^) of the study area was defined as natural foci. Since the maps show where the enzootic plague are more likely to occur and where natural foci probably exist, they can be used to guide surveillance of plague in future.

Generally speaking, human plague cases are directly or indirectly associated with the epizootic plague activity. In Qinghai-Tibetan Plateau, most human plague outbreaks stemmed from contact with infected marmots. We estimated more than 2,000,000 people at the risk of exposure to the enzootic plague. Most risk population are distributed in the east and south of Qinghai-Tibetan Plateau. Our findings can guide the design and spatial targeting of plague prevention and control efforts against human plague infection in Qinghai-Tibetan Plateau. Thereby, limit resource could be appropriately allocated to the a few areas where most human plague cases are most likely to occur.

We got a risk map that shows the full extent of areas of potential natural foci with similar environmental conditions to the observed presence infected host. However, it is important to keep in mind that some unmappable risk factors were not included in our analysis. Natural barriers and other factors may also accidentally obstruct the dispersal of marmots, vector fleas, or *Y. pestis* into the certain areas where habitats are suitable for persistence of natural foci. In addition, epizootic activity may be quite dynamic in the same plague focus in different periods [15,18,42,43]. Temporal variation of climate and other environmental factors may drive the dynamics of epizootic activity in foci. Thus multivariate time series analysis of plague epizootic is required. Finally, with the implementation of the western China development strategy, the traffic transport between Qinghai-Tibetan Plateau and other parts of China has become much more convenient than before, especially since the completion of Qinghai-Tibet Railway in 2006. There will be more and more people such as tourists and transient workers get into Qinghai-Tibetan Plateau in future. The risk of human exposure to enzootic plague and long-distance transmission will increase consequently. Thus the risk of human plague should be further assessed under the scenario of increasing mobility of the population in Qinghai-Tibetan Plateau.

## Conclusions

Maxent was suitable for this 1 km resolution modeling which outputs the distribution probability of the host marmots, its feasibility for other resolution would be tested in the following study. We got a more detailed view of spatial pattern of potential plague natural foci by maximizing training sensitivity and specificity simultaneously. Human-inhabited areas and population at the risk of exposure to enzootic plague could be identified by overlying Chinese population distribution map to the risk map created above. The maps could help public health authorities decide where to perform plague surveillance and take preventive measures in Qinghai-Tibetan Plateau.

## Competing interests

It should be understood that none of the authors have any financial or scientific conflicts of interest with regard to the research described in this manuscript.

## Authors’ contributions

QQ carried out the main modeling progress. JZ drafted the manuscript. LF guided the model building. WZ, LW and HY participated in data interpretation and statistical analysis. HZ and WY participated in data acquisition and coordination. WC and QL was the main designer and final approval of the version to be published. All authors read and approved the final manuscript.

## Pre-publication history

The pre-publication history for this paper can be accessed here:

http://www.biomedcentral.com/1471-2334/14/382/prepub
